# Outcomes of microsurgical vasoepididymostomy using intussusception technique: a systematic review and meta‑analysis

**DOI:** 10.1038/s41598-023-28637-6

**Published:** 2023-02-27

**Authors:** Shou-yang Wang, Yang-yi Fang

**Affiliations:** 1 Department of Urology, Beijing Tiantan Hospital, Capital Medical University, Beijing, 100070 China; 2 Department of Urology, Peking University Third Hospital, Beijing, 100191 China

**Keywords:** Male factor infertility, Urogenital diseases

## Abstract

A systematic review and meta-analysis of microsurgical vasoepididymostomy (MVE) for treating epididymal obstructive azoospermia (EOA) with different intussusception techniques. We conducted a comprehensive literature search using PubMed, Embase, and the Cochrane Central Register of Controlled Trials, retained literature related to obstructive azoospermia or male infertility and vasoepididymostomy, proactively reviewed other relevant literature, supplemented valuable references, and excluded studies that did not use intussusception and where valuable statistical data were difficult to obtain. Event rate and risk ratio (RR) were estimated. Patency rates were investigated. The influence of motile sperms found in the epididymal fluid, anastomotic sides and sites on patency was evaluated. 273 articles were comprised in this analysis, and 25 observational studies were eventually included, with a total patient sample of 1400. The overall mean patency rate was 69.3% (95% confidence interval [CI] 64.6–73.6%; *I*^2^ = 63.735%). We conducted a meta-analysis of the factors affecting patency after microsurgical IVE, finding that the presence of motile sperms in epididymal fluid (RR = 1.52; 95% CI 1.18–1.97%; *P* = 0.001), anastomosing bilaterally (RR = 1.32; 95% CI 1.15–1.50%; *P* < 0.0001) and distally (RR = 1.42; 95% CI 1.09–1.85%; *P* = 0.009) lead to higher patency rates. IVE is an effective treatment for EOA. The presence of motile sperms found in the epididymal fluid, anastomosing bilaterally and distally are significantly correlated with higher patency rates.

## Introduction

Microsurgical vasoepididymostomy (MVE) is an effective technique for epididymal obstructive azoospermia (EOA) to improve male fertility. Since the new century, intussusception vasoepididymostomy (IVE) has become the mainstream mode of MVE, which has a higher surgical patency rate^1,2^. Microsurgical longitudinal intussusception vasoepididymostomy (LIVE) has been the preferred operation for EOA treatment since 2004^3^, and this procedure requires superior surgical skills and meticulous surgical technique^4^.

Evaluating the differences in surgical results with different techniques and finding out the factors affecting surgical results will provide the basis for the decision-making of surgical methods and obtain better surgical results.

This analysis aims to systematically review the evidence for IVE treatment of EOA, explore differences in outcomes among different IVE techniques, and provide a meta-analysis of their effectiveness.

## Methods

### Literature search

This systematic review and meta-analysis were reported following the Preferred Reporting Items for Systematic Reviews and Meta-Analyses (PRISMA) and MOOSE Guidelines for Meta-analyses and Systematic Reviews of Observational studies.

Databases searched included PubMed, Embase, and the Cochrane Central Register of Controlled Trials from inception to March 20, 2022. Exhaustive electronic search was conducted by two independent authors (Wang S. and Fang Y.) using the subject terms ((“azoospermia” [All Fields]) OR (“epididymal obstructive azoospermia [All Fields]”) OR (“epididymal obstruction” [All Fields]) OR (“epididymis obstruction” [All Fields]) OR (“vasoepididymal obstruction” [All Fields]) OR (“infertility” [All Fields]) OR (“fertility” [All Fields])) AND ((“vasoepididymostomy” [All Fields]) OR (“epididymovasostomy” [All Fields])). The two independent authors evaluated the searching results. The senior author (Hong K.) reviewed these articles to ensure their relevance to our study and determined the articles included. The search strategy was initially developed for PubMed and subsequently adapted for the other databases. Event rate and risk ratio (RR) were estimated using a random‑effects model. Heterogeneity was investigated using the Q statistic and *I*^2^ values.

### Inclusion and exclusion criteria and outcome measures

During the screening, we included articles evaluating the effect of vasoepididymostomy on EOA patency rate and those comparing the effect of different factors on patency. We excluded review articles, animal studies, < 10 case series, and a previous meta-analysis. We excluded articles in which IVE was not carried out in treating EOA. There are no language or publication date restrictions for this analysis.

We extracted the following data from the retrieved studies: author names and years of publication, methods of study design, surgical techniques, sample sizes, patients’ and partners’ ages, patency rates, pregnancy rates and follow-up. We also extracted intraoperative information, including the presence or absence of motile sperms in epididymal fluid, anastomotic sides and sites of epididymal.

### Quality assessment

The NIH “Quality Assessment Tool for Before-After (Pre-Post) Studies with No Control Group”, which can be achieved at https://www.nhlbi.nih.gov/health-topics/study-quality-assessment-tools, was used for uncontrolled before-after studies. This tool is composed of 12 items that could be answered as “Yes”, “No”, “Not Reported” or “Not applicable”, and the overall quality of each study could be classified as “Good”, “Fair” or “Poor”. In this analysis, however, the 12th item should always be answered as “Not applicable”, thus the highest quality score theoretically is 11.

### Statistical analyses

RevMan 5.3 (Cochrane Community) and Comprehensive Meta-Analysis V3 (CMA; Biostat) were used for the meta-analysis. SPSS 24.0 (IBM) was used for statistical analysis of quality assessment. Event rates or RR and 95% confidence interval (CI) for dichotomous variables were investigated. Due to the heterogeneity of the included studies, the Mantel–Haenszel random-effects model was used. All *P* values are two-tailed, and *P* < 0.05 was considered statistically significant.

Q statistics were used to test homogeneity between studies. Homogeneity is rejected when the Q statistic *P* value is < 0.10.

The effects of IVE on patency, in total and in different techniques respectively, were shown by the forest plots, which contain a pooled estimate of the effect (event rate or RR) as a dashed vertical line with a diamond at the bottom representing the 95% CI. Individual studies are represented as squares with their CIs, and the weight of each study is represented by the proportion of its corresponding square.

## Results

### Eligible studies

**Figure 1:**
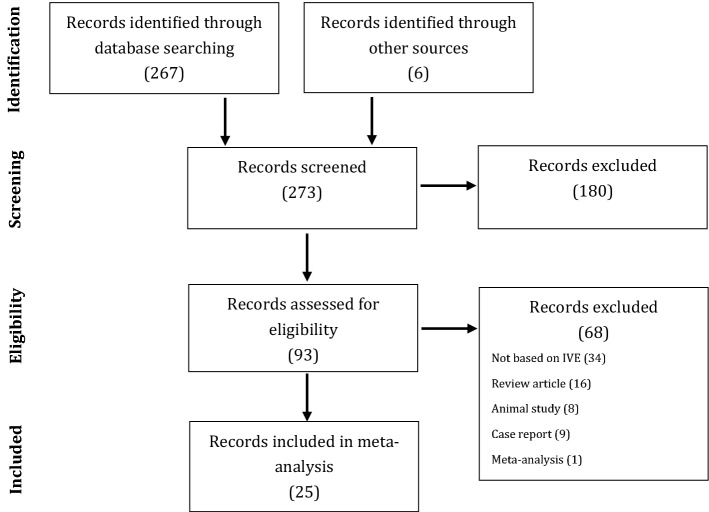
Flow chart depicting the study selection process (PRISMA flow diagram).

Among 273 retrieved articles, 25 met the eligibility criteria and were included in the system review^2,5–28^. A PRISM flow chart describing the results of study identification and selection is shown in Fig. 1.

Among these 25 studies (9 prospective cohort studies and 16 retrospective cohort studies), 1400 patients in the meta-analysis were aggregated qualitatively and quantitatively (Table 1).

**Table 1:** Study and patient characteristics among included studies.

Study	Design	Technique	N	Age (range)	Age of partner (range)	Time for patency, months (range)	Patency (rate, %)	Pregnant rate, %	Follow-up, months (range)	Quality score*
Chan, P. T. et al.^5^	P	3-suture double-armed triangulation	63	39.8 (22–57)	31.8 (24–41)	2.1	53 (84)	40	15.2 (1–36)	8
Schiff, J. et al.^2^	R	3-suture double-armed triangulation	19	N/A	30.7	2.8 (1–11)	16 (84)	46	70.8	9
		2-suture double-armed longitudinal	15		30.4	2.9 (1–6)	12 (80)	44	17.2	
Kumar, R. et al.^6^	P	2-suture double-armed longitudinal	23	30.7 (24–38)	N/A	3.2 (1.5–7)	11 (48)	N/A	7.6 (1.5–30)	5
Ho, K. L. et al.^7^	R	3-suture double-armed triangulation	6	36	30	4 (1–20)	4 (67)	32	15 (4–32)	8
		2-suture double-armed longitudinal	17				9 (53)			
Zhang, G. X. et al.^8^	R	2-suture double-armed longitudinal	42	37	N/A	N/A	30 (71.4)	26.3	> 6–12	7
Kumar, R. et al.^9^	P	2-suture double-armed longitudinal	23	31 (23–40)	N/A	6.6 (3–15)	11 (48)	N/A	11.47 (3–26)	7
Smrkolj, T. et al.^10^	P	3-suture double-armed triangulation	34	34.5 (21–49)	30.4 (19–40)	N/A	21 (63.6)	38.2	N/A	8
Peng, J. et al.^11^	R	2-suture double-armed longitudinal	72	30.4 (21–57)	N/A	N/A	46 (63.9)	N/A	24 (11–45)	6
Peng, J. et al. ^12^	P	2-suture double-armed longitudinal	53	30.9 (22–48)	N/A	4.3 (3–9)	38 (71.7)	33.3	13.5 (4–22)	9
Zhang, H. et al.^13^	R	2-suture double-armed transverse	16	33.0 (21–52)	N/A	N/A	11 (68.8)	37.5	46–63	8
Zhao, L. et al.^14^	R	2-suture single-armed longitudinal	17	30.4	N/A	N/A	10 (58.8)	N/A	N/A	7
Binsaleh, S. et al.^15^	R	2-suture single-armed longitudinal	22	31 (23–47)	25 (21–36)	3 (1–24)	13 (59)	36	18 (6–30)	7
Jiang, H. T. et al.^16^	P	2-suture double-armed transverse	33	31.6 (22–45)	28.5 (20–42)	N/A	29 (87.9)	44.8	22 (9–52)	6
Peng, J. et al.^17^	R	2-suture double-armed longitudinal	53	30.4 (22–48)	27.3 (21–35)	3.6 (3–7)	42 (79.2)	35.8	19.8 (6–43)	9
Zhao, L. et al.^18^ 2015	R	2-suture single-armed longitudinal	39	31.4	29.2	6.2 (1.5–12)	24 (61.5)	38.5	10.3 (2.5–12)	7
Chen, X. F. et al.^19^	P	2-suture single-armed longitudinal	150	28.5 (22–38)	N/A	N/A	108 (72)	38.7	16.5 (4–28)	8
Hong, K. et al.^20^	R	2-suture single-armed longitudinal	62	31 (23–45)	N/A	N/A	41 (66.1)	34.1	8.8 (2–17)	8
Peng, J. et al.^21^	R	2-suture double-armed longitudinal	198	31.0 (20–51)	28.4 (18–42)	3.8 (2–10)	151 (76.3)	40.9	25.3 (12–48)	9
Lyu, K. L. et al.^22^	R	2-suture single-armed longitudinal	59	31.1 (18–42)	N/A	N/A	49 (83.1)	40.7	15.6 (3–33)	8
Wang, S. Y. et al.^23^	R	2-suture single-armed longitudinal	82	30.7	N/A	N/A	59 (72.0)	32.8	19 (12–33)	7
Tang, S. X. et al.^24^	R	2-suture single-armed longitudinal	69	25 (21–42)	N/A	N/A	50 (72.5)	34.0	12.0 (3–29)	7
Shimpi, R. K. et al.^25^	P	2-suture double-armed longitudinal	40	30.2 (24–37)	N/A	N/A	25 (62.5)	15	N/A	7
Tiwari, D. P. et al.^26^	P	2-suture double-armed longitudinal	19	30.1 (22–38)	N/A	N/A	6 (31.6)	5.2	14	6
Liu, N. et al.^27^	R	2-suture single-armed longitudinal	134	32.1 (23–50)	27.2 (20–43)	4.1	74 (55.2)	40.9	17 (3–36)	8
Li, P. et al.^28^	R	2-suture single-armed longitudinal	40	30.4	28.9	3.6	33 (82.5)	51.5	16.9 (12– 23)	9

*R retrospective cohort, *P* prospective cohort, *N/A* not available.

### Quality assessment and publication bias

Table 1 shows the quality score for each article measured by the “Quality Assessment Tool for Before-After (Pre-Post) Studies with No Control Group”. In Statistical analysis by SPSS, the median quality score was 8 (interquartile range 7–8).

**Figure 2:**
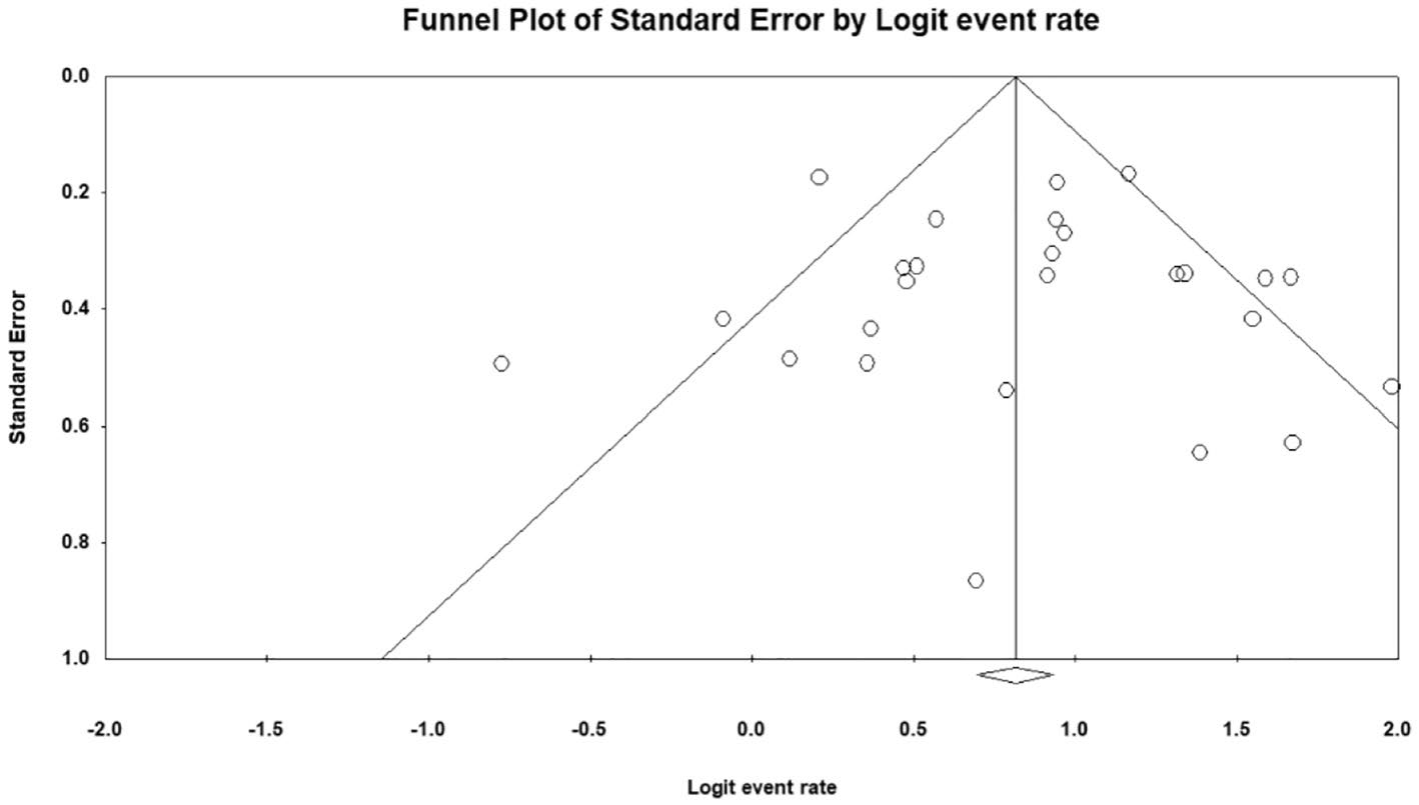
Publishing bias: funnel plots for patency.

The publication bias in this analysis was shown in funnel plots (Fig. 2). There was no publication bias in the overall patency rate (*P* = 0.99, Egger’s test).

### Patency

The definition of patency after IVE varied among all the 25 studies. 9 studies defined patency in different sperm concentrations: > 10^4^ sperms/ml (8) and > 10^6^ sperms/ml (1). In other studies, intact sperm (2), motile sperm (1), or any sperm (9) seen in the ejaculates were considered patency. The definition of patency was not clear in the remaining 4 studies. Natural pregnancy observed is always considered patency.

**Figure 3:**
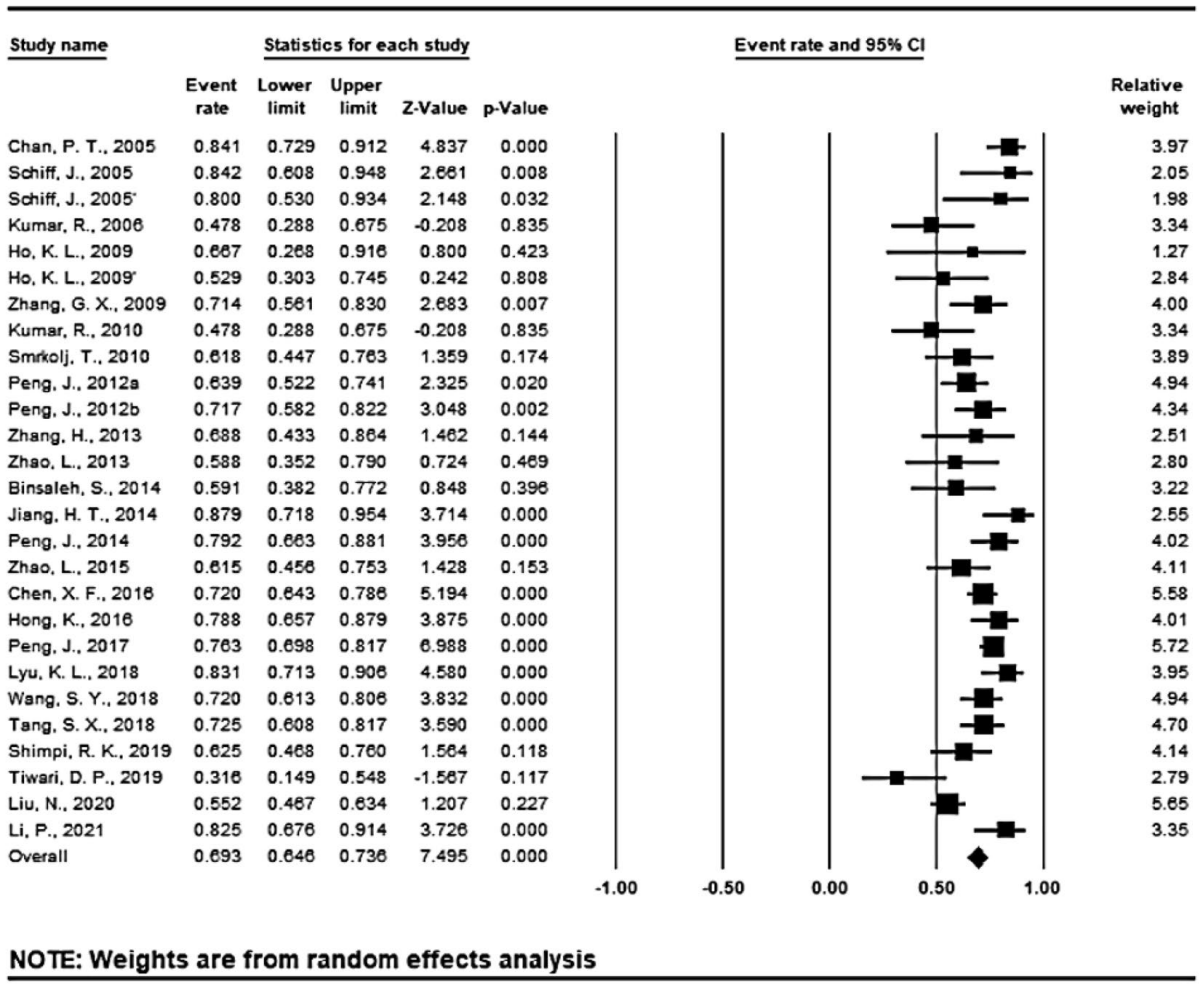
Forest plots of overall patency rate; *CI* confidence interval. Annotation: 2 IVE techniques were reported by Schiff, J. et al.^2^ and Ho, K. L. et al.^7^ respectively, and were statistically analyzed separately in this analysis.

**Figure 4:**
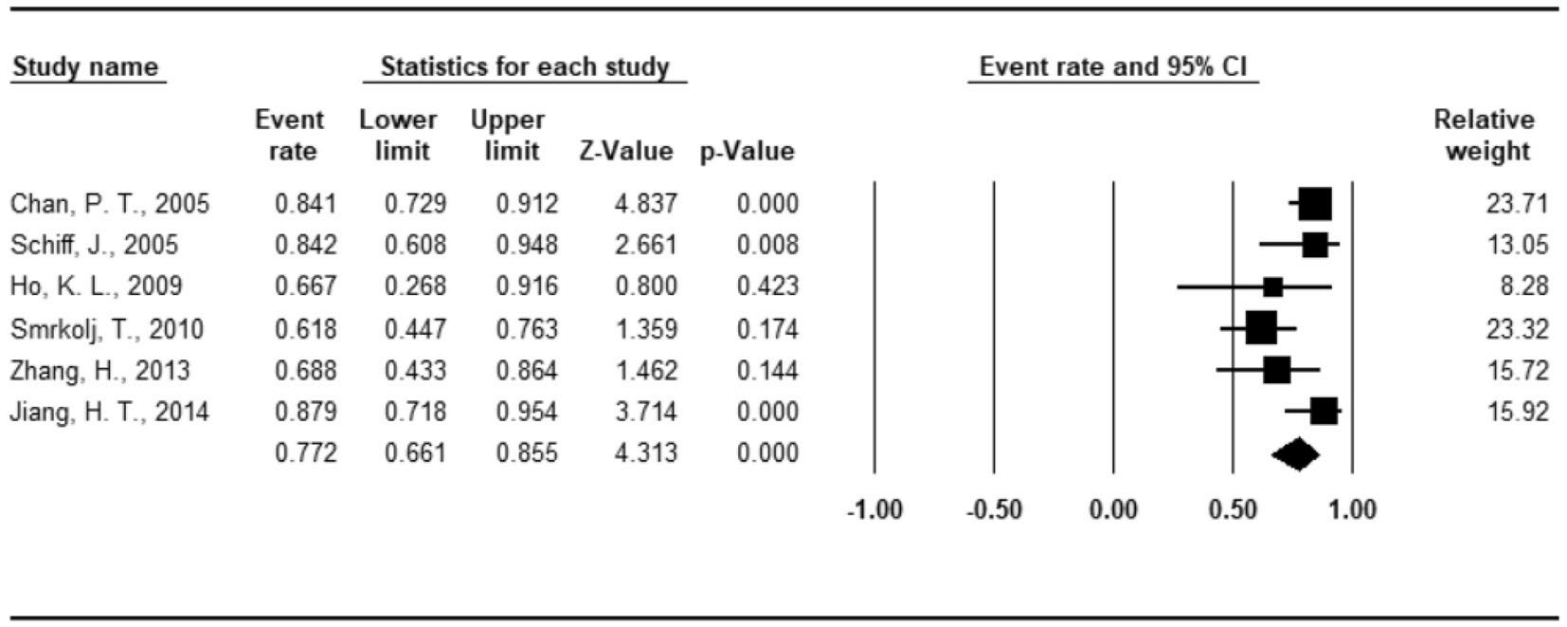
Forest plots of patency rate under 3-suture double-armed triangulation and 2-suture double-armed transverse IVE technique.

**Figure 5:**
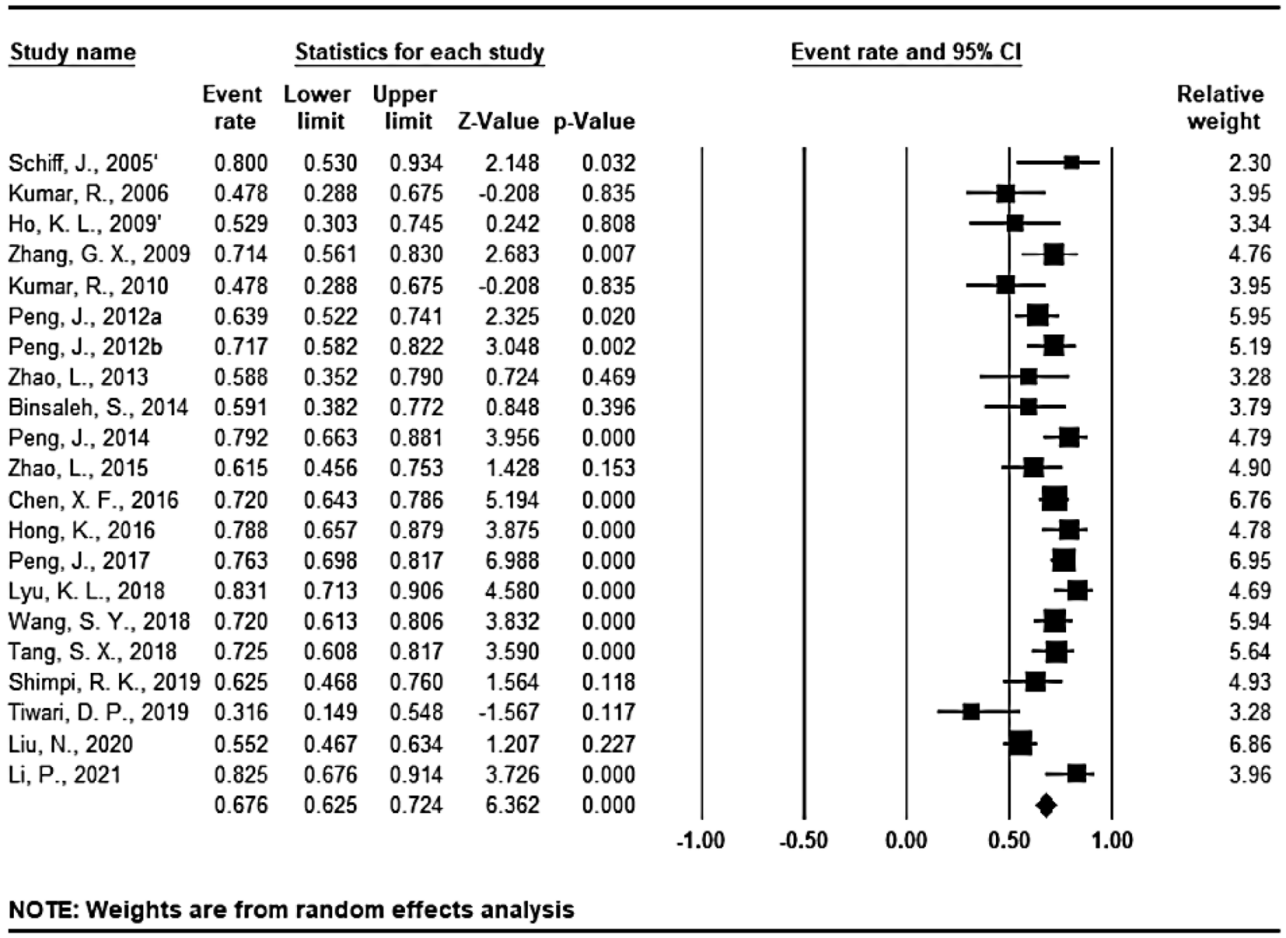
Forest plots of patency rate under LIVE technique.

Patency was analyzed according to different surgical techniques in all the 25 studies (27 sequences) (Figs. 3, 4 and 5). The overall mean patency rate was 69.3% (95% CI 64.6–73.6%; *I*^2^ = 63.735%), 3-suture double-armed triangulation and 2-suture double-armed transverse IVE mean patency rate was 77.2% (95% CI 66.9–85.5%; *I*^2^ = 48.155%) (75.9 and 80.0% respectively). LIVE mean patency rate was 67.6% (95% CI 62.5–72.4%; *I*^2^ = 65.258%).

### Pregnancy

Although “pregnancy rates” were reported in 20 studies (Table 1), the definition of “pregnancy rates” and methods of computation were different, and the duration of follow-up varied widely from study to study: “Natural pregnancy & Pregnancy/All” in 9 studies, “Natural pregnancy & Pregnancy/Patency” in 7 studies, “Natural pregnancy or ART & Pregnancy/All” in 5 studies, “Natural pregnancy or ART & Pregnancy/Patency” in 2 studies, and "Pregnancy method unspecified” in 1 study. Moreover, most studies did not specify the method of determining pregnancy. So, “pregnancy rates” based on these 20 studies were not the same variable and had no statistical value indeed.

### Factors affecting patency

**Figure 6:**
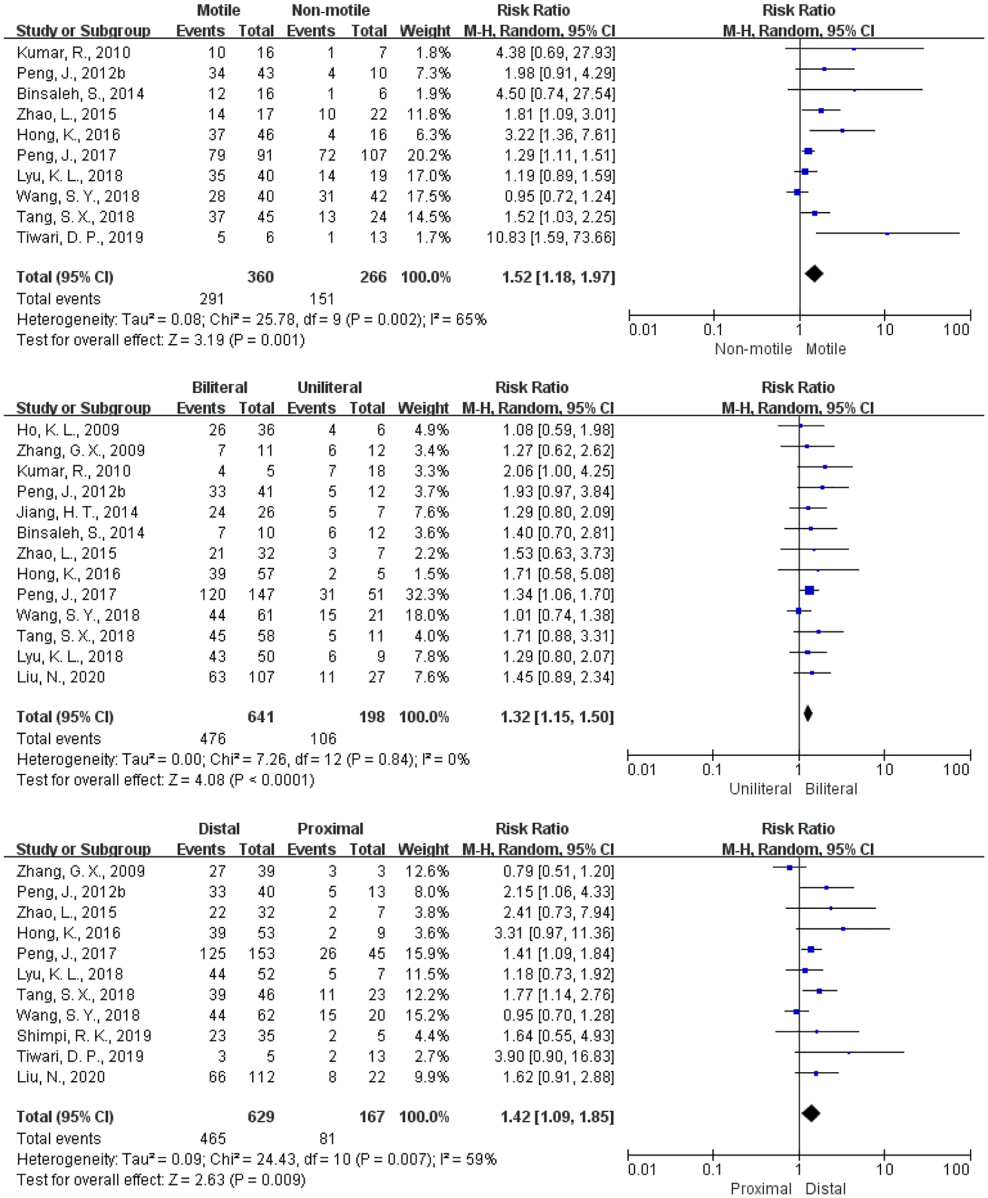
Forest plots of comparison of different factors affecting patency rate: the presence of motile vs. non-motile sperms found in epididymal fluid, anastomosis bilateral vs. unilateral, and anastomosing distal vs. proximal respectively.

Factors affecting patency after IVE were analyzed and presented by forest plots (Fig. 6).

In 10 studies involving 626 patients, those with the presence of motile sperms found in epididymal fluid showed higher patency rate (RR = 1.52; 95% CI 1.18–1.97%; *P* = 0.001).

13 studies involving 839 patients analyzed the patency rate according to anastomosis sides (unilateral or bilateral). Compared with the unilateral group, patients in the bilateral group exhibited higher patency rate (RR = 1.32; 95% CI 1.15–1.50%;* P* < 0.0001).

The effect of anastomotic sites on patency rate was analyzed in 11 studies involving 796 patients. Distal anastomosis leads to a higher patency rate than proximal one (RR = 1.42; 95% CI 1.09–1.85%;* P* = 0.009).

## Discussion

Several systematic evaluations have preliminarily confirmed the safety and efficacy of MVE in the treatment of EOA^1,29^. IVE has gradually replaced other surgical techniques for EOA treatment since the beginning of this century, and remains to be one of the most technically challenging urological surgery. In this analysis, including 25 studies (9 prospective cohort studies and 16 retrospective cohort studies) involving 1400 patients, the efficacy of IVE was analyzed, and the total patency rate was 69.3%, which confirmed that this surgical technique had good efficacy in treating patients with EOA, and was proved to have a higher patency rate than previous MVE surgical techniques^1^.

Currently, LIVE is considered to be the preferred operation for treating EOA^3^.Contrary to common belief, we found that other IVE techniques (3-suture double-armed triangulation and 2-suture double-armed transverse IVE) can achieve a higher patency rate than LIVE (77.2 vs. 67.3%). We tried to explain this result as follows. The triangulation technique, which is not a mainstream technique nowadays, was reported in earlier studies. As was described in the earlier studies, in the cases where the epididymal tubule was too small to accommodate three micro sutures, the 2-suture technique, especially the longitudinal technique, should be used. Therefore, the triangulation technique showed a higher patency rate because of the bias of its patient population with a relatively wider epididymal tubule. It is similar to the 2-suture transverse technique.

Several studies have suggested that motile sperm detected in epididymal fluid, bilateral, and distal epididymal anastomosis can improve the patency rate. In this analysis, we confirmed that motile sperms found in epididymal fluid (80.8% [291/360] vs. 56.8% [151/266]), bilateral anastomosis (74.3% [746/641] vs. 53.55% [106/198]) and distal anastomosis (73.9% [465/629] vs. 48.5% [81/167]) lead to obvious higher patency rate. According to this conclusion, urologists can make more appropriate surgical decisions under certain objective conditions, to improve the patency rate and promote the rehabilitation of male fertility. Bilateral IVE should be considered in all circumstances that are possible in EOA. Moreover, IVE may lead to natural pregnancy, avoiding the risks of assisted reproductive technology (ART) to both female partners and fetuses^30^.

At the same time, we recognize several limitations of this analysis. First of all, we found differences or ambiguities in the definitions of “patency rate” and “pregnancy rate” in previous studies. Especially for “pregnancy rate”, the same name means different parameters indeed. Then, this analysis only included observational studies and most of the studies (16) were retrospective. In addition, several included studies were performed in same centers (Peng, J. et al.^11,12,17,21^; Zhao, L. et al.^14,18^) which may cause unpredictability bias. Increasing the sample size can further eliminate the influence of bias. Combined with the result that “different IVE techniques obtain different patency rates” found in this analysis, which is contrary to popular belief, it is feasible to design randomized controlled trial (RCT) studies of different surgical techniques on the patency rate of IVE. Finally, other factors which may affect patency rates were not systematically reviewed and analyzed limited by the number of studies.

## Conclusions

Microsurgical vasoepididymostomy using intussusception technique proved to have good patency rates. Thus, IVE is effective in improving the fertility of men from EOA. The presence of motile sperms in perioperative epididymal fluid, and anastomosing bilaterally and distally are significantly correlated with higher patency rates in IVE.

## Data Availability

All data generated or analyzed during this study are included in this published article and its supplementary information files.
